# Molecular Mechanism of Citrate Efflux by the Mitochondrial Citrate Transporter CT in Filamentous Fungus *Mucor circinelloides* WJ11

**DOI:** 10.3389/fmicb.2021.673881

**Published:** 2021-05-14

**Authors:** Wu Yang, Shiqi Dong, Junhuan Yang, Hassan Mohamed, Aabid Manzoor Shah, Yusuf Nazir, Xiuzhen Gao, Huirong Fan, Yuanda Song

**Affiliations:** ^1^ Colin Ratledge Center for Microbial Lipids, School of Agriculture Engineering and Food Science, Shandong University of Technology, Zibo, China; ^2^ Tianjin Key Laboratory of Radiation Medicine and Molecular Nuclear Medicine, Institute of Radiation Medicine, Chinese Academy of Medical Sciences & Peking Union Medical College, Tianjin, China; ^3^ Department of Botany and Microbiology, Faculty of Science, Al-Azhar University, Assiut, Egypt; ^4^ Department of Food Sciences, Faculty of Science and Technology, Universiti Kebangsaan Malaysia, Bangi, Malaysia

**Keywords:** *Mucor circinelloides*, citrate carrier, mitochondrial citrate transporter, citrate efflux, transport activity

## Abstract

The mitochondrial citrate transporter (MCT) plays an important role in citrate efflux from the mitochondria in eukaryotes, and hence provides a direct correlation between carbohydrate metabolism and lipid synthesis. Our previous studies on transporters confirmed the presence of two MCTs (TCT and CT) in oleaginous *Mucor circinelloides* WJ11 associated with high lipid accumulation. However, the molecular mechanism of citrate efflux from the mitochondria by MCT in *M. circinelloides* is still unclear. To study the citrate transport mechanism of CT, the citrate transporter gene was expressed in *Escherichia coli*, and its product was purified. The citrate transport activity of the protein was studied in CT reconstituted liposomes. Our results showed high efficiency of CT for [^14^C] citrate/citrate exchange with *K*_m_ 0.01 mM at 25°C. Besides citrate, other molecules such as oxaloacetate, malate, fumarate, succinate aconitate, oxoadipate, isocitrate, and glutamate also promote citrate transport. In addition, the *ct* overexpression and knockout plasmids were constructed and transferred into *M. circinelloides* WJ11, and the mitochondria were isolated, and the transport activity was studied. Our findings showed that in the presence of 10 mM malate, the mitochondria of *ct*-overexpressing transformant showed 51% increase in the efflux rate of [^14^C] citrate, whereas the mitochondria of the *ct*-knockout transformant showed 18% decrease in citrate efflux compared to the mitochondria of wild-type WJ11. This study provided the first mechanistic evidence of citrate efflux from the mitochondria by citrate transporter in oleaginous filamentous fungus *M. circinelloides*, which is associated with high lipid accumulation.

## Introduction

*Mucor circinelloides* is an oleaginous filamentous fungus that has the ability to synthesize long-chain polyunsaturated fatty acids (LC-PUFAs), particularly the omega-6 LC-PUFA γ-linolenic acid (18:3n6). This fatty acid has been proven to play an important role in the physiological functions of human, such as anti-inflammation, anticardiovascular disease, antitumor, antidiabetes, etc. (Manku et al., 1984; Hagi et al., 2010). Because of its importance in the study of fungal lipid biochemistry, *M. circinelloides* has been developed and widely used as a model microbe, whereas its genomic sequence and genetic tools are now readily available (Ratledge and Wynn, 2002). *M. circinelloides* has also been widely explored for its various useful biotechnological applications including removal of metal ions, adsorbing contaminated materials (Saenge et al., 2011), production of functional polysaccharide and biodiesel (Kamoun et al., 2019; Zininga et al., 2019), and for terpenoid production (Nagy et al., 2019). In addition, recent studies of our research group found that this fungus has the potential to produce stearidonic acid (18:4, n-3), dihomo-γ-linolenic acid (20:3, n-6), medium-chain fatty acids (Hussain et al., 2019), and various metabolic intermediate in the biosynthetic pathway to produce eicosapentaenoic acid (Yang et al., 2020a).

Similar to other oleaginous microorganisms, nitrogen limitation and excess carbon strategies were often employed to induce the intracellular accumulation of lipids (Ratledge and Wynn, 2002; Xia et al., 2011). Nitrogen depletion led to a series of enzymatic reactions, leading to the accumulation of citrate in the mitochondria (Liang and Jiang, 2013; Tang et al., 2013). The citrate was then transported into the cytosol and metabolize by ATP-citrate lyase to form acetyl-CoA, which is an important precursor for lipid biosynthesis (Wynn et al., 2001). Nevertheless, as citrate cannot diffuse through the mitochondrial membrane, the involvement of the mitochondrial citrate transport system is then necessary to facilitate the transport process (Manku et al., 1984; Iacobazzi and Infantino, 2013). This led to the identification of MCT [described in previous literature as mitochondrial citrate carrier (CiC)]. MCT belongs to the mitochondrial carrier family (MC) that has a specific character of three times tandemly repeated 100-residue domain, containing two hydrophobic segments and a signature sequence motif PX [D/E]XX [K/R]X [K/R] (20–30 residues) [D/E]GXXXX [W/Y/F][K/R]G (PROSITE PS50920, PFAM PF00153, and IPR00193; Palmieri, 1994; Porcelli et al., 2014). To date, the atomic resolution three-dimensional structures of only one member (ADP/ATP carrier) of MC family have been determined (Pebay-Peyroula et al., 2003). MCT was initially discovered and purified from human and animal livers, and numerous subsequent studies have revealed its mechanism of mitochondrial citrate transport (Dolce et al., 2014; Palmieri and Monné, 2016) . MCT catalyzes an electroneutral, obligatory exchange of the dibasic form of a tricarboxylic acid (citrate, isocitrate, and *cis*-aconitate) for another tricarboxylate/H^+^, a dicarboxylate (malate, succinate, and malonate), or phosphoenolpyruvate (Bisaccia et al., 1990, 1993). Through isolation of intact mitochondria (LaNoue and Schoolwerth, 1979) or reconstruction of recombinant proteins in liposomes (Zara et al., 1996), numerous studies have been conducted to study the selectivity and kinetic properties of the substrate transported by MCT (Bisaccia et al., 1990), as well as its relationship between the primary structure and function of proteins (Kaplan and Mayor, 1993; Ma et al., 2006; Remani et al., 2008; Aluvila et al., 2010). By determining the level of gene expression (Tamano et al., 2013; Wasylenko et al., 2015), protein activity and promoter function, the role of MCT has been thoroughly studied. It was found that the changes in the expressing of MCT have direct effects on its activity and transport efficiency, which, in turn, has a close relation to lipid biosynthesis (Gnoni et al., 2009; Iacobazzi and Infantino, 2013; Iacobazzi et al., 2013; Damiano et al., 2015). For example, the citrate concentration in the mitochondria of oleaginous yeast was three to four times higher than that of non-oleaginous yeast. Furthermore, the efflux rate of citrate in the mitochondria of oleaginous yeast was 2.5 times higher than that of non-oleaginous yeast. Comparison of *K*_m_ value showed that the citrate transporter of oleaginous yeast could transport citrate more effectively when the malate concentration in the cytoplasm was lower (Evans et al., 1983a,b). It is found that the human and rat MCT promoters have been regulated by multiple transcription factors including sterol regulatory element (SRE), stimulating protein 1 region (Sp1), fork head box A (FOXA), peroxisome proliferator-activated receptor-responsive element (PPRE) motif, nuclear factor Y(NF-Y) site, and E-box-like site-binding sequences (Dolce et al., 2014). And the long-chain fatty acyl-CoA has a feedback inhibition on citrate transport (Evans et al., 1983b).

The identification and characterization of substrate specificity and transport mechanism of *Saccharomyces cerevisiae* and *Yarrowia lipolytica* MCT members named Ctp1p and Yhm2p have been studied (Kaplan et al., 1995; Scarcia et al., 2017; Yuzbasheva et al., 2019). Nevertheless, a very limited study has been conducted to understand its function and regulation in oleaginous filamentous fungi (Yang et al., 2019). Thus, in this study, the citrate transport mechanism of the mitochondrial transporter CT from *M. circinelloides* WJ11 was studied for the first time by [^14^C]-labeled citrate transport analysis using the purified protein and isolated mitochondria.

## Materials and Methods

### Strains, Media, and Culture Conditions

*Escherichia coli* BL21 (DE3) competent cells were used for *ct* gene heterologous expression. *E. coli* cultivation media and their conditions have been previously described (Deininger, 1990). *M. circinelloides* WJ11 (CCTCC no. M 2014424; China Center for Type Culture Collection) was used as recipient strain for *ct* gene overexpression and knockout in transformation experiments. The culture conditions of the recombinant strain were as follows: *M. circinelloides* cultures were initiated by inoculation of approximately 10^6^–10^7^ spores/ml into 150-ml K&R medium (1-L flask equipped with baffles) and incubated in an incubator shaker at 28°C, 150 revolutions/min (rpm) for 24 h. Then, the cultures were inoculated into 2-L bioreactors (BioFlo/CelliGen115, New Brunswick Scientific, Edison, NJ, United States) containing a 1.5-L modified K&R medium (Kendrick and Ratledge, 1992). The bioreactor was controlled following our previous work (Yang et al., 2019). Culture samples of each strain were collected at 72 h for extraction (Evans et al., 1983a).

### Bioinformatics Analysis of MCT Genes

Identification of putative mitochondrial transporter genes in WJ11 was done through gene annotations using different databases such as Kyoto Encyclopedia of Genes and Genomes, National Center for Biotechnology Information (NCBI), non-redundant proteins, protein families (Pfam), and transporter classification database (TCDB). The phylogenetic tree was constructed by MEGA 6.0 based on the sequences, which were found in the gene annotation of *M. circinelloides* WJ11 that may be encoding mitochondrial transporters (Yang et al., 2020b). According to the predicted function in TCDB, one transporter has been found, named CT [also known in the literature (Gnoni et al., 2009) as the citrate carrier, CiC] encoded by scaffold00129.3, which might be involved in mitochondrial citrate transportation. Based on the searching results of NCBI-PubMed database, the amino acid sequences of citrate transporters of yeasts that resemble *Mucor* and mitochondrial transporter family member whose crystal structures have been determined were aligned with our sequenced CT. Sequences homology analyses were performed using Pairwise Sequence Alignment and then aligned with ClustalW and ESPript.

### Transport Activity Determination of CT Reconstituted Liposomes

#### Heterologous Expression and Purification

The complete *ct* gene sequence was optimized (according to *E. coli* codon usage), synthesized, and subcloned into target vector pET30a(+) for expression. Plasmid pET30a(+)-*ct* was constructed on the basis of cloning strategy: pET30a-NdeI-ATG-*ct*-Histag-Stop codon-*Hin*dIII-pET30a.

One hundred nanograms of plasmid DNA was added into BL21 (DE3) strain and mixed gently. Heat shock method was used for transformation, and then the plate was incubated at inverted position at 37°C overnight. The pET30a-*ct* recombinant strain BL21 (DE3) was inoculated into 5,052 autoinduction medium (Studier, 2005) containing kanamycin and cultured at 37°C. When the OD_600_ reached about 1.2, cell culture was induced with IPTG at 15°C for 16 h, and then the cells were harvested by centrifugation. Cell pellets were resuspended with lysis buffer (50 mM Tris, 150 mM NaCl, 5% glycerol, pH 8.0) followed by sonication for 10 min. The precipitate was then dissolved using urea. Denatured protein was obtained by one-step purification using Ni-column. Target protein was renatured and sterilized by 0.22-μm filter. Purified CT was solubilized in 1x phosphate-buffered saline (PBS), pH 7.4; 10% glycerol; and 0.5 M l-arginine. The concentration was determined by Bradford protein assay with bovine serum albumin as standard. The samples of whole cell lysate, supernatant, and debris were analyzed using sodium dodecyl sulfate-polyacrylamide gel electrophoresis (SDS-PAGE) and Western blot. The protein purity and molecular weight were determined by standard SDS-PAGE along with Western blot confirmation. The primary antibody for Western blot is anti-His antibody (GenScript, cat. no. A00186).

#### Reconstitution of CT Liposomes and Their Transporting Activity Assay

The liposomes were prepared by adding 232, 58, and 94 mg of soybean lecithin, cholesterol, Tween 80, respectively, in 15-ml mixture of chloroform:methanol (3:1). This mixture was then poured into a round-bottom flask and then rotationally evaporated for 30 to 60 min at 50°C followed by the addition of 20 ml of 20 mM phosphate buffer. The resulting solution was placed in the ultrasonic bath for 10 min.

The solubilized recombinant protein was diluted three times with a buffer containing 3% Triton X-114 (wt/vol), 20 mM Na_2_SO_4_, and 10 mM piperazine-1,4-bisethanesulfonic acid (PIPES, pH 7.0) and reconstituted into liposomes. The reconstituted liposome system was designed as follows: 1% TritonX-114, ultrasound-prefabricated liposome, 20 mM PIPES, 0.8 mg cardiolipin, and water replenish to finally 700 μl. These components were blended gently, and the mixture was recycled 13 times passed through a hydrophobic chromatography column (Bio-Rad Beads SM-2). Columns were pre-equilibrated with 10 mM PIPES (pH 7.0) and the substrates. The substrate here is to be embedded in a liposome that is used to exchange citrate (Palmieri et al., 1995; Monné et al., 2018; Yuzbasheva et al., 2019). Except for the passages through column that were carried out at room temperature, all other operations were performed at 4°C. The amount of purified protein reconstructed into the liposome was determined by the method as described by Porcelli et al. (2014), and approximately 11.2% of the protein was added to the reconstructed mixture.

The Sephadex G-75 columns were pre-equilibrated with buffer (10 mM PIPES and 50 mM NaCl, pH 7.0) to remove the substrates from the protein proteoliposomes. Transport at 25°C was initiated by adding radioactive [^14^C]citrate (PerkinElmer Life Sciences) to substrate loaded (exchange) or empty (uniport) proteoliposomes. The reaction was terminated by adding 20 mM pyridoxal-5'-phosphate (PLP), which inhibits the activity of several MCT completely and rapidly (Marobbio, 2003; Marobbio et al., 2006). In controls, according to the “inhibitor-stop” method, the inhibitor was added together with the [^14^C]citrate at the beginning (Palmieri et al., 1995). Finally, Sephadex G-75 was used to remove the external radioactivity, and the radioactivity of the protein liposomes was measured by Liquid Scintillation Analyzer (PerkinElmer, Tri-carb 4910TR; Palmieri et al., 1999; Yuzbasheva et al., 2019). *K*_m_ values were calculated by linear regression analysis of the transport results.

### Mitochondrial Transport Properties of CT Mutants in WJ11

#### Construction of Overexpression and Knockout Recombinant Mutants

The plasmids used in this study were pMAT2081-*ct* and pMAT2060-*ct* for *ct* gene overexpression and knockout, respectively, and were constructed by our research group (Yang et al., 2019, 2020b). Both pMAT2081-*ct*, pMAT2060-*ct*, and the empty plasmid pMAT2075 were transformed to MU760, which was the uridine auxotrophic strain derived from WJ11, and the albino colonies were selected (Rodríguez-Frómeta et al., 2012).

#### Isolation of Mitochondria

*M. circinelloides* were grown for 72 h, and mycelium was filtered on preweighed Whatman no. 1 filter paper. The resulted mycelium was washed twice with buffer (50 mM Tric/HCl and 1.2 M sorbitol, pH 6.5); 1.5 g of lysing enzymes (containing β-glucanase, cellulase, protease, and chitinase activities; Sigma) was added to 250 ml stabilizing buffer (1.2 M MgSO_4_ and 10 mM KH_2_PO_4_ at pH 6.0). The filtered mycelium in buffer was incubated for 3 h at 30°C with speed of 100 rpm for protoplast preparation (Yusuf et al., 2018). The resulting mixture was subjected to gradient centrifugation 500, 1,000, and 2,000 *g* for 5 min individually to remove cell impurity substances and 11,000 *g* for 20 min for mitochondria isolation (BestBio kit BB-36017). Isolated mitochondria were washed and weighted and were resuspended in the buffer; the final concentration of mitochondria was adjusted to 1 mg/ml and stored at 0°C for further study.

#### Mitochondrial Viability Assay

To detect the mitochondrial viability, NADH and fumarate were added to the mitochondria suspension: 100 μl NADH (0.5 mmol), 20 μl fumarate (7 mmol), 70 μl 1x PBS buffer (pH 7.4), and 10 μl mitochondria. The absorbance of biochemical reaction mixture were taken by microporous plate absorbance spectrophotometer (Bio-Rad xMark™) at a wavelength of 340 nm after 20 min incubation using the enzymatic kinetic method (Evans et al., 1983b).

#### Measurements of Mitochondria Transporting Activity

The mitochondrial suspension was preincubated to “load” the mitochondria with [^14^C]citrate in a 30°C water bath and gently shaken for 3 min before adding the substrate (Evans et al., 1983b). The reaction is started by simultaneous addition of malate or α-ketoglutarate and stopped by rapid centrifugation. Uptake of citrate was determined by measuring the incorporation of [^14^C]citrate radiolabel into mitochondrial pellets and the disappearance of [^14^C]citrate radiolabel from the incubations; 10 mM substrates were added at the same time to give a final volume of 1.0 ml. The reactions were stopped after 5 min by rapidly separating the mitochondria from the incubation mixture using the same conditions of centrifugation.

### Statistical Analysis

SPSS 16.0 was used to analyze all statistical data of three independent values. The results were presented as mean ± SD. The differences were statistically significant at *p* < 0.05.

## Results

### Comparative Analysis of MCTs From *M. circinelloides* WJ11 With Citrate Transporters From Other Fungi

**Figure 1:**
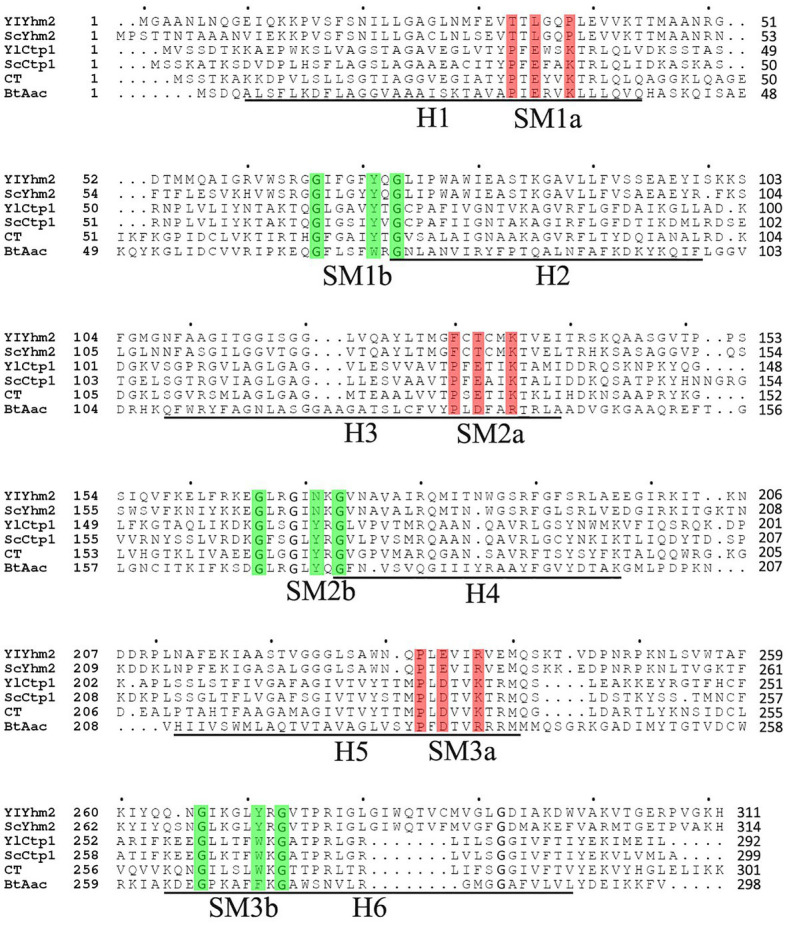
Amino acid sequences of CT were aligned with its homologs in *Saccharomyces cerevisiae*, *Yarrowia lipolytica*, and the sequences of ADP/ATP carrier. The sequences of six transmembrane helices (H1–H6) from the ADP/ATP carrier are underlined. Odd transmembrane helix in red part a and even transmembrane helix in green part b represent 3-fold repeated signature motif (SM) characteristic of the MC family proteins. YlYhm2 and YlCtp1 were citrate transporters from *Y. lipolytica*; ScYhm2 and ScCtp1 were citrate transporters from *S. cerevisiae*, and BtAac1 was the ADP/ATP carrier from *Bos taurus*, which was the member of MC family whose three-dimensional structures have been determined.

In the previous work of our group, the genome of *M. circinelloides* WJ11 has been annotated (Yang et al., 2019). It revealed that 51 genes were encoding for possible mitochondrial transporter family proteins. Among these, scaffold00129.3 was annotated as citrate transporter, and scaffold0069.38 was annotated as tricarboxylic acid transporter, encoded by genes named *ct* and *tct*, respectively. These annotations indicated that both genes may be involved in citrate transport in *M. circinelloides* WJ11. According to the phylogenetic tree of all 51 mitochondrial transporters in *M. circinelloides* WJ11 and evolutionary status presented in our previous work (Yang et al., 2019, 2020b), CT protein located in a branch containing a 2-oxodicarboxylate carrier, three succinate/fumarate mitochondrial transporters, adjacent branches are ADP/ATP carriers and RNA splicing protein, respectively. The amino acid sequences of the MCT members Yhm2p and Ctp1p from *S. cerevisiae* and *Y. lipolytica*, respectively, and MC member Aac (ADP/ATP carrier named BtAac) from *Bos taurus* were used for multiple sequence alignment with CT from *M. circinelloides* WJ11. CT showed 50.7, 48.6, 25.2, 26, and 24.3% identities with YlCtp1p, ScCtp1p, YlYhm2p, ScYhm2p, and BtAac, respectively. The multiple sequence alignment (Figure 1) indicated that CT has the unique characteristics of all members of the mitochondrial transporter family and with highly conserved domain structure of MCT family. The underlined sequences H1–H6 were six transmembrane helices from the ADP/ATP carrier. Odd transmembrane helix in red part and even transmembrane helix in green part b represent threefold repeated signature motif (SM) characteristic of the MC family proteins. The red color indicates the PX [D/E] XX [K/R] X [K/R] sequences, and the green color indicates the [D/E] GXXXX [W/Y/F] [K/R] G sequences. CT has three Mtc domains pfam00153 (amino acid residues 10–104, 106–199, and 209–296) blasted by NCBI-CDD (Yang et al., 2019). Therefore, *ct* was selected for the further experimental identifications.

### Citrate Transporting Activity of the Recombinant CT

**Figure 2:**
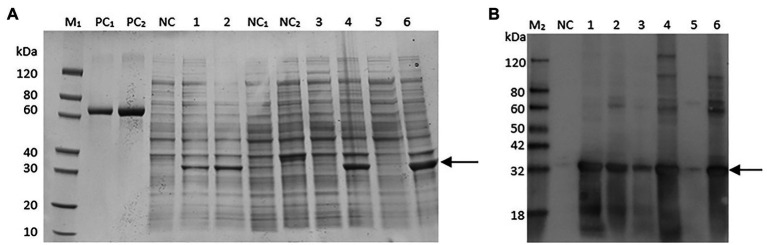
Sulfate-polyacrylamide gel electrophoresis (SDS-PAGE) **(A)** and Western blot **(B)** analysis for *ct* cloned in pET30a (+) and expressed in BL21 (DE3) strain. Markers (bovine serum albumin: PC, protein marker: M1, Western blot marker: M2) are shown in the left column. Lanes 1, 3, and 5 represent cell lysate, supernatant of cell lysate, and debris of cell lysate, respectively, of *Escherichia coli* cells containing the expression vector inducted for 16 h at 15°C. Lanes 2, 4, and 6 represent cell lysate, supernatant of cell lysate, debris of cell lysate, respectively, of recombinant *E. coli* cells induced by IPTG for 4 h at 37°C.

To study the activity of the mitochondrial citrate transporter protein CT, the *ct* gene was inserted into pET30a(+), transformed, and expressed in *E. coli* BL21 (Figure 2A).

The expressed protein CT was accumulated as inclusion bodies, which was purified by one-step purification using the Ni column. The final concentration of the purified protein was 0.75 mg/ml, and the yield of purified protein was about 3.75 mg/L of culture. The identity of the recombinant protein was confirmed by Western blot analysis (Figure 2B), which showed that the molecular weight of the purified protein was about 32 kDa, which is consistent with the calculated value (32 kDa).

**Figure 3:**
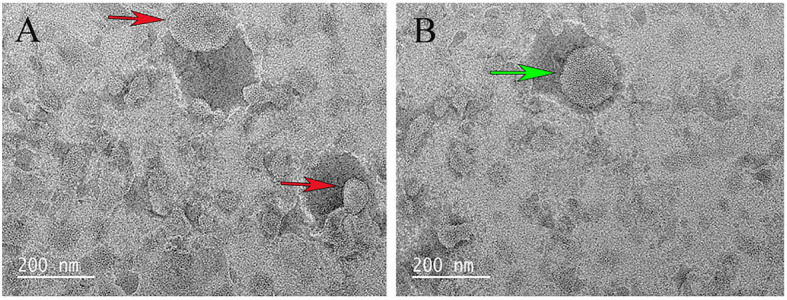
Scanning electron microscopy examination of liposome. **(A)** The red arrow indicates the liposome. **(B)** The green arrow indicates the liposome after CT reconstitution.

The purified recombinant protein CT was reconstituted into liposomes, and its transport activity was tested on various potential matrices by homo-exchange (with the same substrate inside and outside the proteoliposomes). Compared with pure liposomes, the surface of liposomes containing proteins was somewhat not smooth (Figure 3).

**Figure 4:**
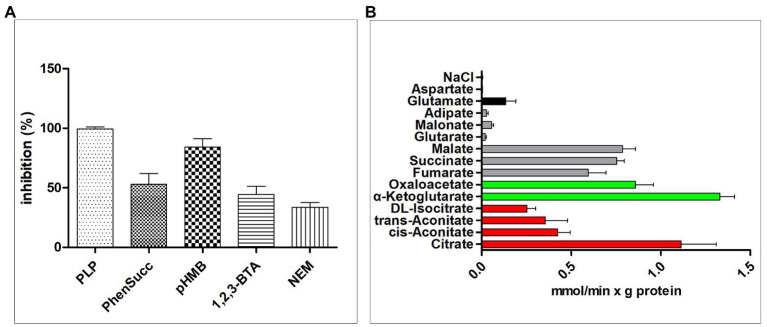
Transport properties of recombinant CT. **(A)** Effect of inhibitors on the citrate/citrate exchange by CT. Liposomes were reconstituted with CT and preloaded internally with 10 mM citrate. Transport was initiated by adding 0.01 mM [^14^C]citrate and terminated after 2 min. The concentrations of the inhibitors were 20 mM (PLP, pyridoxal 5'-phosphate), 2 mM (phesucc, phenylsuccinate), 0.1 mM (pHMB, *p*-hydroxymercuribenzoate), 2 mM (1,2,3-BTA, 1,2,3-benzenetricarboxylate), 1 mM (NEM, *N*-ethylmaleimide). **(B)** Dependence of CT transport activity on the internal substrate. Reconstructed liposomes containing CT were preloaded internally with a variety of substrates (20 mM). Transport was initiated by adding 0.01 mM [^14^C]citrate and terminated after 1 min. The values are means ± SEM of at least three independent experiments in duplicate for each internal substrate investigated. Red: tricarboxylic acids. Green color: α-ketodicarboxylic acids. Gray color: dicarboxylic acids. Black color: other compounds. Error bars represent SDs (*n* = 3).

The effects of some MCT inhibitors on the [^14^C]citrate/citrate exchange reaction catalyzed by reconstituted CT were also examined (Figure 4A). This activity was inhibited strongly by PLP, so PLP was used as an inhibitor for reaction termination. The homo-exchange activity of CT at internal and external concentrations of 10 μM and 10 mM of [^14^C]citrate and citrate, respectively, was inhibited by PLP.

To study the substrate specificity of CT in detail, the initial uptake rate of 0.01 mM [^14^C]citrate of the CT liposome preloaded with a variety of potential substrates was determined (Figure 4B). The highest absorption activity of [^14^C]citrate in proteoliposomes was achieved by internal citrate, α-ketoglutarate, malate, oxaloacetate, succinate, and fumarate. [^14^C]Citrate is also exchanged, at a less extent, with internal isocitrate and oxoadipate. The transport affinity (*K*_m_) and the specific activity (*V*_max_) values for the citrate/citrate exchange at 25°C were 0.01 mM and 2.32 mmol/min per gram of protein, respectively.

### Functional Identifications of CT in the Mitochondria of the Transformants

**Figure 5:**
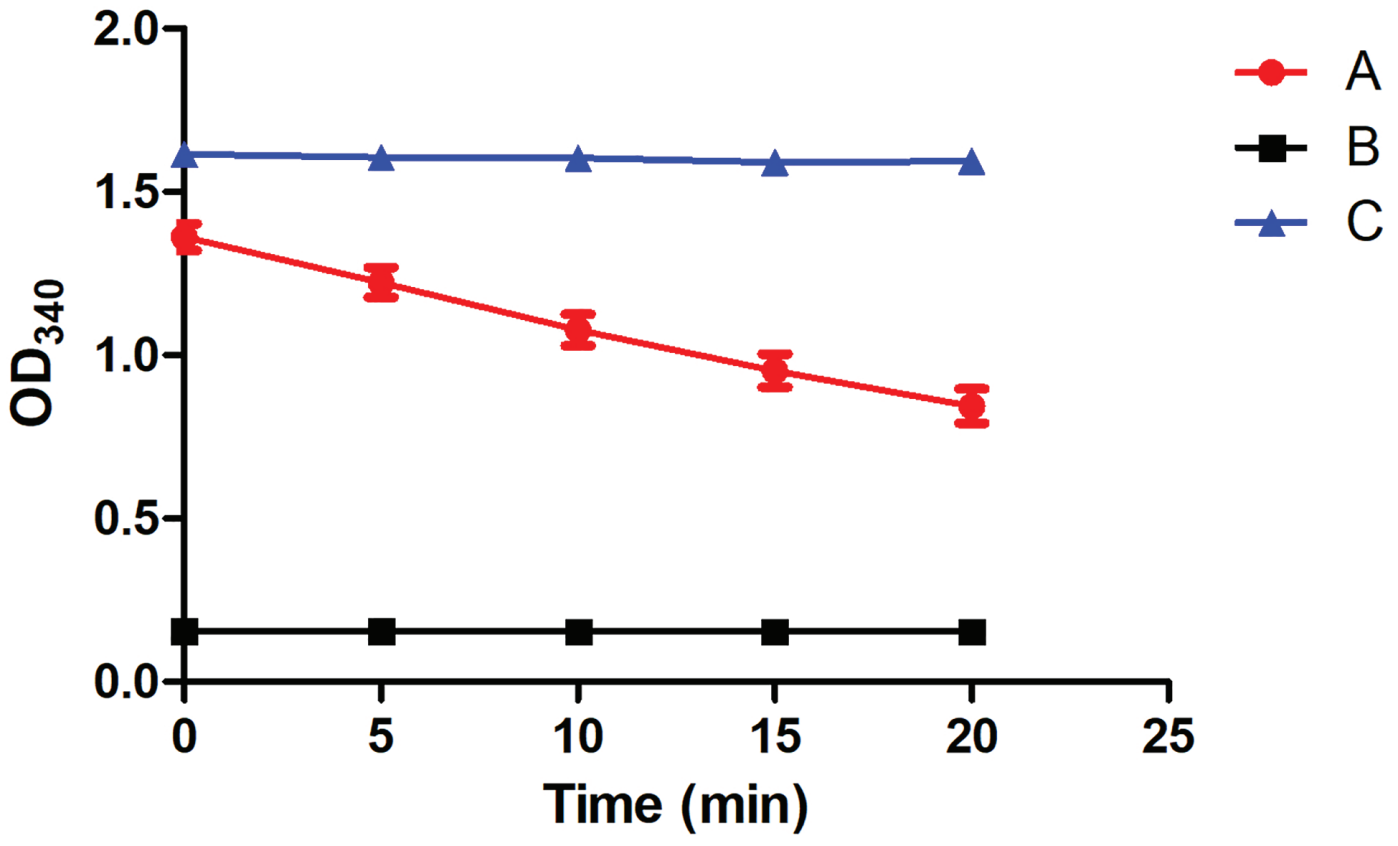
Detection of the mitochondrial activity by the respiratory chain. (A) A complete system containing the extracted mitochondria. (B) Negative control, NADH not included. (C) Positive control, fumarate not included. Error bars represent SDs (*n* = 3).

To verify whether CT participates in the transport of citrate in *M. circinelloides* WJ11, the *ct*-overexpression and -knockout transformants of *M. circinelloides* WJ11 were constructed, and the target *ct* fragment has been integrated into the WJ11 genome and expressed (data not shown). After cultivation in a medium with an excess glucose and limited nitrogen for 72 h, the mycelia were collected and prepared for mitochondria isolation. Microscopic examination of mitochondria was carried on after staining using Janus Green B, and the stained mitochondria were green in color, which indicated that the mitochondria isolated from the fungus were intact. As shown in Figure 5, the mitochondrial viability assay results showed that the NADH/NAD^+^ ratio of respiration activity of the mitochondria was between 0.8 and 1.4, indicating that mitochondrial preparations were viable.

**Figure 6:**
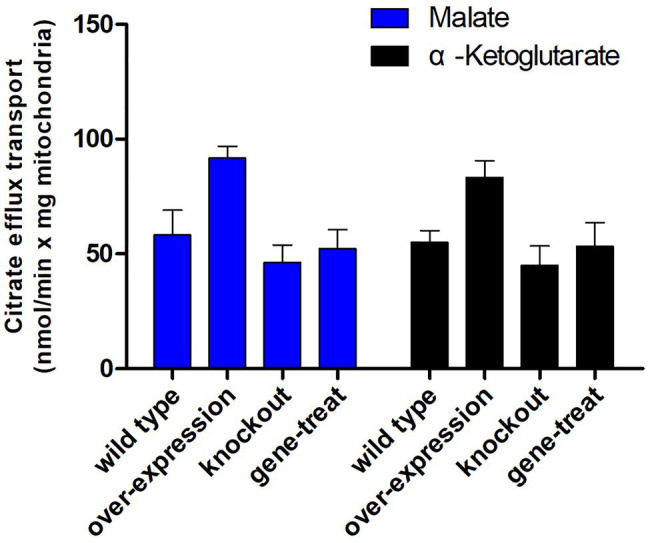
Mitochondrial transport activity of *ct*-transformants. Mitochondrial transport efficiency were represented of mitochondria from the wild-type transformants, overexpressed, knockout, and gene repair, respectively. Mitochondria were preloaded with 0.01 mM [^14^C]citrate. Blue and black columns were substrates (10 mM malate and 20 mM α-ketoglutarate, respectively) outside of mitochondria. Error bars represent SDs (*n* = 3).

Furthermore, the mitochondria were incubated with 0.01 mM [^14^C]citrate, and the efflux transport activities were detected. The results as shown in Figure 6 demonstrated that in the presence of 10 mM malate and 10 mM α-ketoglutarate outside the mitochondria, mitochondria from *ct*-overexpressing transformants showed a 51% increase in the rate of [^14^C]citrate outflow compared with the mitochondria from the wild-type strain, whereas the rate with *ct*-knockout transformants was 18% lower than that of the wild type. In addition, after the complementation of the *ct* in the knockout strain, the citrate exchange system was fully restored. These results demonstrated that mitochondrial transporters transported citrate; manipulation of *ct*-expressing level affected the efficiency of mitochondrial transport of citrate. The outflow efficiency of mitochondria from *ct*-overexpressing transformant reached 95 mmol/min per milligram of mitochondria, which was much higher than the control.

## Discussion

During carbohydrate utilization, most eukaryotes use citrate produced by the tricarboxylic acid (TCA) cycle in the mitochondrial matrix to generate acetyl-CoA in the cytoplasm for the synthesis of important compounds such as fatty acids, sterols, and *N*-acetylglucosamine (Aluvila et al., 2010). Citrate is a key substrate for the generation of energy and a modulator of multiple enzymatic activities. In the mitochondria, citrate is oxidized *via* the TCA cycle and oxidative phosphorylation, and in the cytoplasm, citrate inhibits glycolysis and restores oxaloacetate and acetyl-CoA, whereas acetyl-CoA serves as the precursor for *de novo* lipid synthesis (Owen et al., 2002). Because of huge potential of microbes in lipid especially PUFA production, *M. circinelloides* is usually used as a model organism for the study of the mechanism of lipid accumulation. The systematic study on basic biochemistry of lipid accumulation in *M. circinelloides* is under process (Vellanki et al., 2018). However, the regulation mechanism of citrate transport from the mitochondria into the cytosol in fungi has not been fully investigated.

In this study, for the first time, we identified and determined the functional characteristics of citrate transporter across the mitochondrial membrane of *M. circinelloides*. The amino acid sequences of the MCT members include Yhm2p, Ctp1p, and Aac obtained from *S. cerevisiae*, *Y. lipolytica*, and *B. taurus* respectively, and were used for multiple sequence alignment with protein CT from *M. circinelloides* WJ11. The sequence alignment results of protein CT from WJ11 having the unique characteristics of all members of the MCT family represented 50.7, 48.6, 25.2, 26, and 24.3% identities with YlCtp1p, ScCtp1p, YlYhm2p, ScYhm2p, and BtAac, respectively. The previous study by Saier et al. (2016) exhibited the sequence alignment of SbCtp1 and SbYhm2 with their *S. cerevisiae* homologs and the *B. taurus* ADP/ATP translocase and validated the classification of both SbCtp1 and SbYhm2 in the MCT member (TCDB 2.A.29; Saier et al., 2016). Additionally, the identical amino acids of Yhm2p showed only 21 and 22% with Ctp1p and CTP, respectively (Castegna et al., 2010); comparing with other work, it can be concluded that CT belongs to the MCT family.

Traditionally, the recombinant transporting proteins were reconstituted into liposomes to identify and characterize their transport characteristics (Palmieri and Monné, 2016). In our study, CT from *M. circinelloides* WJ11 was cloned, and the protein was purified and reconstituted in liposomes for activity assay. No exchange activities were detected by [^14^C]citrate/citrate exchange when CT was inactivated by PLP. Notably, the highest absorption rate of [^14^C]citrate in liposomes was achieved by citrate, α-ketoglutarate, malate, oxaloacetate, succinate, and fumarate, while the absorption of [^14^C]citrate was slow in the presence of isocitrate and oxoadipate. The transport affinity (*K*_m_) and the specific activity (*V*_max_) values for the citrate/citrate exchange at 25°C were 0.01 mM and 2.32 mmol/min per gram of protein, respectively. Similar to our results, the activity of MCT of [^14^C]citrate/citrate exchange was inhibited by the SH-blocking reagents, such as PLP, *p*-hydroxy mercury benzoate, and partially by the alkylating reagent *N*-ethylmaleimide (Monné et al., 2018; Yuzbasheva et al., 2019). Other studies (Quagliariello and Palmieri, 1971; Prezioso et al., 1972) had demonstrated that [^14^C]citrate/citrate exchange is also inhibited by 1,2,3-benzenetricarboxylate. Based on *K*_m_ values, our results indicated that the affinity of CT for citrate is significantly higher than those previously reported (Genchi et al., 1999; Zara et al., 2003); it was found that the *K*_m_ values of *S. cerevisiae* CiC1 and CiC2 bacterially expressed proteins for citrate were 0.36 and 0.16 mM, respectively. Besides, the *K*_m_ values of Yhm2p for citrate and oxoglutarate were approximately 0.2 and 1.2 mM, respectively (Castegna et al., 2010). However, Yhm2p cannot transport malate and isocitrate and is not inhibited by 1,2,3-benzenetricarboxylate. In a recent study in *Y. lipolytica*, citrate transporter YlYhm2p was demonstrated to transport citrate with exchange of α-ketoglutarate, oxaloacetate, succinate, fumarate, *cis*-aconitate, *trans*-aconitate isocitrate, oxoadipate, and malate, and the *K*_m_ values for the citrate/citrate exchange at 25°C were 0.15 mM per gram of protein (Yuzbasheva et al., 2019).

In our previous work, we have constructed the mitochondrial citrate transporter overexpression mutants in low-lipid-producing strain *M. circinelloides* CBS 277.49, and it produced increased amounts of lipids (Yang et al., 2019). In this work, we isolated the mitochondria from *ct*-overexpression and -knockout transformants of high-lipid producing *M. circinelloides* WJ11, and the citrate transporting activities of these mitochondria were determined. Our results showed a 51% increase in [^14^C]citrate outflow rate in the mitochondria of *ct*-overexpression transformant, whereas 18% decrease in that of *ct*-knockout transformant, compared to that of the wild-type strain, in the presence of 10 mM malate or 10 mM α-ketoglutarate outside the mitochondria. Moreover, the citrate efflux efficiency of mitochondria from *ct*-overexpressing transformants reached 95 mmol/min per milligram of mitochondria, which is much higher than the control. This increase in citrate transporting activity in the mitochondria of *ct*-overexpression transformant is associated with the increase in lipid accumulation (data not published) and indicated that CT plays a vital role in mitochondria citrate/malate transport and hence in lipid accumulation. However, citrate transporting activity of the mitochondria of the *ct*-knockout mutants was not greatly affected as the *ct*-overexpressing mutants; this suggested that another citrate transporter TCT may take the role of citrate transporting when CT is not available; indeed, lipid accumulation in *ct*-knockout mutant is not significantly decreased (data not published).

So far, there is very limited information about citrate transporting activity in the mitochondria of the oleaginous fungi; only one early report by Ratledge (Evans et al., 1983c) showed that malate was the preferred substrate for citrate efflux, and moreover, citrate transporting activity of the mitochondria from the oleaginous yeast was much higher than that from the non-oleaginous yeast, suggesting that the citrate/malate shuttle powered by the citrate transporters in oleaginous yeast plays an importing role in lipid accumulation. This is more or less in agreement with our research results.

Our findings demonstrated that the CT is a key citrate transporter in *M. circinelloides* WJ11, which significantly contributed to the mitochondrial output of citrate in the cytosol for lipid metabolism. The proposed mechanism of citrate transport is sequential (Bisaccia et al., 1993), which implies the essential presence of a counter substrate for citrate efflux. The citrate/malate antiport fulfills important metabolic demands (Palmieri and Pierri, 2010). In this context, with the obtained mitochondria from overexpression of genomic sequence encoding MCT members, our attempt to determine the functional characterization of the MCT is a powerful tool for expanding our knowledge on this issue.

In yeasts, citrate transported from mitochondria into the cytoplasm may be converted into α-ketoglutarate under the action of NADP^+^-dependent isocitrate dehydrogenase, and the resultant α-ketoglutarate is transported back to mitochondria to complete the cycle (Dolce et al., 2014). CT transporter belongs to the MCT family, and it can regulate the citrate efflux from mitochondria to cytosol, as demonstrated in *Y. lipolytica* (Yuzbasheva et al., 2019). In *S. cerevisiae* and *Y. lipolytica*, two MCT members have been identified (Kaplan et al., 1995; Scarcia et al., 2017; Yuzbasheva et al., 2019). There were also two genes (encoding CT and TCT) in the genome of *M. circinelloides* that may be related to citrate transport. It was observed that the citrate transporter of *M. circinelloides* has very high affinity for its substrate and transports citrate from the mitochondria to cytosol with high efficiency, thus promoting the synthesis of fatty acids by increasing cytosolic citrate.

## Conclusion

The results of this study highlighted the importance of *ct* gene in *M. circinelloides* WJ11. As one of the MCTs in the fungus, CT transports citrate efficiently with high activity. Furthermore, overexpression of *ct* in the fungus significantly increased citrate transport in the mitochondria, which in turn increased lipid accumulation in this fungus. Taken together, our results suggested that CT plays an important role in citrate transport and lipid accumulation in *M. circinelloides*. Our study improved our current knowledge about citrate transport in oleaginous fungi.

## Data Availability Statement

The original contributions presented in the study are included in the article/supplementary material, further inquiries can be directed to the corresponding author.

## Author Contributions

WY planned the experiments, carried out the experimental work, and generated the figures. SD, XG, HF, and JY did the additional experimental work and participated in the writing of the article. WY and SD contributed equally to this paper as first authors. HM, YN, and AS participated in the writing of the article. YS supervised the work and participated in the writing of the article. All authors contributed to the article and approved the submitted version.

### Conflict of Interest

The authors declare that the research was conducted in the absence of any commercial or financial relationships that could be construed as a potential conflict of interest.
